# Synthetic CT Generation of the Pelvis in Patients With Cervical Cancer: A Single Input Approach Using Generative Adversarial Network

**DOI:** 10.1109/access.2021.3049781

**Published:** 2021-01-08

**Authors:** ATALLAH BAYDOUN, KE XU, JIN UK HEO, HUAN YANG, FEIFEI ZHOU, LATOYA A. BETHELL, ELISHA T. FREDMAN, RODNEY J. ELLIS, TARUN K. PODDER, MELANIE S. TRAUGHBER, RAJ M. PASPULATI, PENGJIANG QIAN, BRYAN J. TRAUGHBER, RAYMOND F. MUZIC

**Affiliations:** 1 Department of Radiation Oncology, University Hospitals Cleveland Medical Center, Cleveland, OH 44106, USA; 2 Department of Biomedical Engineering, Case Western Reserve University, Cleveland, OH 44106, USA; 3 School of Artificial Intelligence and Computer Science, Jiangnan University, Wuxi 214122, China; 4 Jiangsu Key Laboratory of Media Design and Software Technology, Jiangnan University, Wuxi 214122, China; 5 Department of Radiology, School of Medicine, Case Western Reserve University, Cleveland, OH 44106, USA; 6 Department of Radiation Oncology, School of Medicine, Case Western Reserve University, Cleveland, OH 44106, USA; 7 Department of Radiation Oncology, Penn State Cancer Institute, Hershey, PA 17033, USA; 8 Philips Healthcare, Cleveland, OH 44143, USA; 9 Department of Radiology, University Hospitals Cleveland Medical Center, Cleveland, OH 44106, USA

**Keywords:** Cervical cancer, computed tomography, deep learning, generative adversarial network, magnetic resonance imaging, U-Net

## Abstract

Multi-modality imaging constitutes a foundation of precision medicine, especially in oncology where reliable and rapid imaging techniques are needed in order to insure adequate diagnosis and treatment. In cervical cancer, precision oncology requires the acquisition of ^18^F-labeled 2-fluoro-2-deoxy-D-glucose (FDG) positron emission tomography (PET), magnetic resonance (MR), and computed tomography (CT) images. Thereafter, images are co-registered to derive electron density attributes required for FDG-PET attenuation correction and radiation therapy planning. Nevertheless, this traditional approach is subject to MR-CT registration defects, expands treatment expenses, and increases the patient’s radiation exposure. To overcome these disadvantages, we propose a new framework for cross-modality image synthesis which we apply on MR-CT image translation for cervical cancer diagnosis and treatment. The framework is based on a conditional generative adversarial network (cGAN) and illustrates a novel tactic that addresses, simplistically but efficiently, the paradigm of vanishing gradient vs. feature extraction in deep learning. Its contributions are summarized as follows: 1) The approach –termed sU-cGAN-uses, for the first time, a shallow U-Net (sU-Net) with an encoder/decoder depth of 2 as generator; 2) sU-cGAN’s input is the same MR sequence that is used for radiological diagnosis, i.e. T2-weighted, Turbo Spin Echo Single Shot (TSE-SSH) MR images; 3) Despite limited training data and a single input channel approach, sU-cGAN outperforms other state of the art deep learning methods and enables accurate synthetic CT (sCT) generation. In conclusion, the suggested framework should be studied further in the clinical settings. Moreover, the sU-Net model is worth exploring in other computer vision tasks.

## I. INTRODUCTION

In the current era of precision medicine, magnetic resonance (MR) imaging emerged as a key element of oncological diagnosis and staging [1], especially for the female pelvis for which computed tomography (CT) images preclude uterus substructures delineation or tumorous tissue discrimination [2]. Consequently, MR is considered as the imaging modality of choice in gynecological cancers in general and in cervical cancer in particular [2]–[4], as it produces images with exquisite soft tissue contrast, provides detailed multiparametric structural and functional radiological data, and requires no x-ray exposure to the patient, [1]. Nevertheless, no current MR-only radiation therapy workflow can be routinely adopted in clinical practice due to multiple factors. At a logistic level, MR requires longer acquisition time than CT, which increases patient discomfort and machine and MR technologists time expenses [5]. Also, MR images do not provide electron density information needed for dose calculations of the radiation therapy plan and for attenuation correction for Positron Emission Tomography (PET)/MR [6]. However, most of the MR-based radiation therapy workflows requires the acquisition of at least an initial planning CT before initiation of the radiotherapy treatment. Subsequently, the MR acquired throughout the radiation therapy treatment timeline can be registered to the planning CT, and electron density information can be retrieved [7]. Nonetheless, this approach remains sub-optimal as dosimetric accuracy can be affected by MR to CT registration imperfections [8]–[11].

To overcome the need for CT acquisition, a multitude of synthetic CT (sCT) generation methods have been designed, experimentally studied, and applied in clinical research [12]. These methods are classically divided into atlas-based, tissue-based, and voxel-based techniques [13]. In atlas-based methods, a library consisting of a previously collected MR-CT pair is used. Each newly-acquired MR volume is registered to its best matching MR volume from the library and the registration field is then applied to the library CT images to produce an sCT for the new patient [14]. Atlas-based methods are subject to deformable registration artifacts, notably in areas with altered anatomy due to tumor growth or surgical void [15], [16]. As for tissue-based methods, the image volume is first divided into tissue classes such as air, fat, and bone. Then, each tissue class is assigned a Hounsfield Unit (HU) value [12]. Tissue-based methods are dependent on manual segmentation and, similarly to atlas-based methods, do not operate at a voxel-level. Voxel-based methods overcome the limitations of atlas-based and tissue-based methods and offer the advantage of applying an MR image intensity to CT HU transformation at a voxel level. This approach was initially based on statistical modeling [17], thresholding [18] or clustering [19], but more recent research is being focused on deep learning [20] given its convenient ability of automatic feature extraction, correlation, and combination.

Breakthroughs in computational imaging over the last decade enabled a significant acceleration of the radiation therapy workflows in general, and particularly of PET/MR-based workflows. Ideally, a conveniently automated PET/MR-based workflow should be based entirely on a single MR sequence for diagnosis, quantitative PET/MR attenuation correction, auto-contouring, and radiation therapy planning. Practically, such workflow has not yet been reported. In cervical cancer, for example, diagnosis would require a T2-weighted, Turbo Spin Echo-Single Shot (TSE-SSH) MR sequence [3], [4], however, neither sCT generation methods using T2-weighted, TSE-SSH MR sequence as input nor auto-contouring have been validated for the female pelvis.

In this manuscript, we present a new deep learning framework for sCT generation for the female pelvis. Our method leverages the generative adversarial network (GAN) image synthesis potential with the U-Net features extraction capacity. Compared to the previously published studies, the novelties of this manuscript are summarized as follows:

1. We introduce the sU-cGAN model that entails a shallow U-Net (sU-Net) with an encoder/decoder depth of 2, as the generator of a conditional GAN (cGAN) network. Given a simplified structure of its generator, sU-cGAN exhibits a lower number of trainable parameters when compared to the commonly used cGAN networks.
2. While most of the previously published articles used multiple MR sequences as input features, sU-cGAN employs a single channel input model by only requiring the heavily T2-weighted, MR sequence; it has not been studied for the female pelvis sCT generation yet.
3. Despite structure simplification and a single channel input, we demonstrate that sU-cGAN enables accurate and rapid sCT generation via an exhaustive, contour-based comparative analysis.
4. By complementing our previously reported auto-contouring report in cervical cancer [21], this study constitutes a novel approach for PET/MR-based attenuation correction and radiation therapy planning in patients with cervical cancer, where the complete workflow (radiological diagnosis, quantitative PET/MR attenuation correction, auto-contouring, and radiation therapy planning) can be solely based on a single MR sequence, i.e. T2-weighted, TSE-SSH.

In Section II of this manuscript, we briefly review the basics of the commonly used MR imaging features as well as U-Net and GAN networks. We then introduce in Section III the proposed sU-cGAN model. In Sections IV and V, we present the experimental setup and discuss the comparative analysis. Lastly, conclusions and future directions are drawn in Section VI.

## II. RELATED WORK

### A. MR INPUT FOR sCT GENERATION

For MR-only radiotherapy and PET/MR attenuation correction, most of the voxel-based sCT production methods used conventional T1-, T2- or Dixon-derived sequences as input [12]. T1-weighting is achieved via applying short time of echo (TE) and time of repetition (TR), while T2-weighting is achieved by applying long TE and TR [22]. As for the Dixon sequence, it takes advantage of chemical shift effects in order to yield in-phase (IP) and opposed-phase (OP) images [23]. Dixon water and fat images can then be created by adding and subtracting the IP and OP images [23]. Therefore, the end result of the Dixon sequence is the production 4 interrelated images: IP, OP, water, and fat.

The usual choice of conventional T1-, T2-, or Dixon-derived sequences as input for sCT generation algorithms derives essentially from two factors: 1) data availability, as conventional T1 and T2 sequences are the most widely used in clinical radiology [22], and Dixon sequences are used in the currently available PET/MR systems for attenuation correction [24]. 2) Dixon sequences allow the use of up to 4 channels as input, which empowers the features extraction capacity of the sCT generation methods and potentially improves the overall accuracy [25]. However, conventional T1 and T2 sequences usually require longer acquisition time than CT, which is a source of discomfort to the patient and leads to geometric distortions in MR images [5].

**FIGURE 1.**
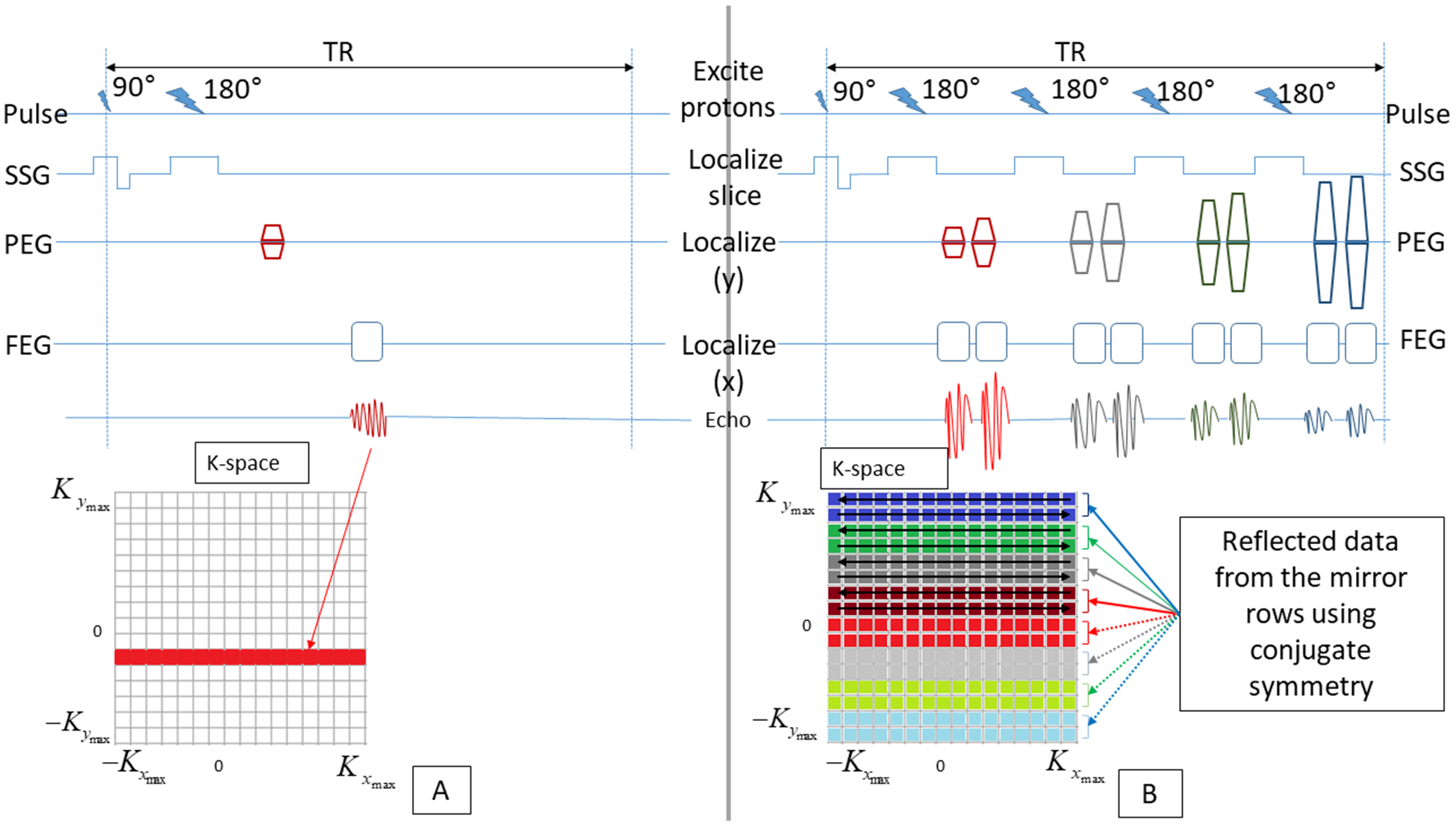
Conventional Spin Echo (a) and TSE-SSH (b). The dark-colored upper rows in K-space in (b) are acquired. The clear-colored lower rows with dotted arrows (b) are filled in K-space using conjugate symmetry. Full arrows to the acquired K-space rows in (b) were not represented for image simplification. SSG = slice select gradient, PEG = phase encoding gradient, FEG = frequency encoding gradient.

Compared to the conventional spin echo, the TSE-SSH enables the acquisition of the complete K-space data in a single TR [26] by applying multiple phase encoding gradients of increasing amplitude during a single TR so that multiple echoes are generated [27] and only half of the K-space needs to be sampled (Fig. 1(b)) [27]. As a result, the acquisition time is greatly shortened, which reduces the geometric distortion and minimizes the breathing and motion artifacts [27]. As such, T2-weighted TSE-SSH MR imaging was included in the recommendations of the International Federation of Gynecology and Obstetrics (FIGO) [3] and the European Society of Urogenital Radiology [4] as it combines the advantage of soft tissue differentiation with the T2 contrast and the geometric fidelity with the TSE-SSH acquisition scheme.

### B. GAN FRAMEWORK

The GAN framework was introduced in 2014 by Goodfellow *et al.* [28], and has been eminently exploited and developed in different areas of computational imaging in general, and in image synthesis in particular [29]. In its original form, the framework consisted of a generator *G*(·) able to generate a synthetic image *G*(*z*) when given a random noise *z* as input. *G* competes with a discriminator *D*(·) whose task is to differentiate whether a given input is a measured *x* or synthetic *G*(*z*) image. *G* and *D* are trained simultaneously in a min-max game fashion, where *G* is attempting to produce realistic data that misleads *D* in its classification task, while *D* is optimizing its capacity of synthetic vs. measured image discrimination. Mathematically, the GAN loss function *L*_*GAN*_ can be designated as follows:

(1) $$L_{GAN}\left(D,G\right)=E_{x\sim p_{\text{data}}\left(x\right)}\left[\text{log }D\left(x\right)\right]+E_{z\sim p_{z}\left(z\right)}\left[\text{log}\left(1-D\left(G\left(z\right)\right)\right)\right]$$

In order to accomplish a class-oriented image synthesis, Mirza *et al.* adjusted the GAN model to include a conditional class *c* such as image modality or category [30]. This framework was labeled as conditional GAN (cGAN), and its loss function *L*_*cGAN*_ can be designated as follows:

(2) $$L_{\text{cGAN}}\left(D,G\right)=E_{x\sim p_{\text{data}}\left(x\right)}\left[\text{log }D\left(x|c\right)\right]+E_{z\sim p_{z}\left(z\right)}\left[\text{log}(1-D\left(G\left(z|c\right)\right)\right]$$

In 2016, Isola *et al*. [31], [32] adapted the cGAN model to the task of supervised image to image translation by making the following changes: 1) The input image *y* was considered to be itself the conditional class; and 2) The noise was considered to be embedded in the input image *y* and was counteracted by applying several layers of dropout at training and testing time [31]. With the above modifications, the cGAN loss function in the case of supervised image to image translation Λ_*cGAN*_ can be written as:

(3) $$L_{\text{cGAN}}\left(D,G\right)=E_{x,y}\left[\text{log }D\left(x|y\right)\right]+E_{x,y}\left[\text{log}(1-D\left(G\left(y\right)\right)\right]$$

Since then, the cGAN framework for image to image translation has been widely applied in the medical literature, notably for pelvic sCT generation. In 2018, Maspero *et al.* generated a pelvic sCT via a cGAN framework using MR Dixon training data from 32 patients having prostate, rectal or cervical cancer [33]. Using a similar approach, Brou Boni *et a*l. reported sCT generation from T2-weighted MR images in 19 male patients with prostate or rectal cancer [34]. In addition, Fetty *et al.* also used T2-weighted MR images to compare the performance of different cGAN generators across different MR magnetic field strengths [35]. To the best of our knowledge, cGAN-based pelvis sCT generation via T2-weighted TSE-SSH has not yet been studied.

### C. U-NET

In a landmark manuscript published in 2015, Ronneberger *et al.* devised - for the initial purpose of semantic segmentation - a deep learning network named U-Net given its symmetrically arranged encoding and decoding pathways [36]. Due to its exceptional ability of feature extraction, even in the settings of limited data, the U-Net model was also studied for image synthesis. In fact, Isola *et al.* had adopted a U-Net architecture as generator in their cGAN framework for image to image translation [31]. While Ronneberger *et al.* had used an encoder/decoder depth of 3 [36], Isola *et al.* executed their experimental analysis with a U-Net encoder/decoder depth of 8, yielding a total of 50 × 10^6^ trainable parameters [31], [32]. To counteract this computational inconvenience, Bass *et al.* performed image synthesis using a convolutional capsule GAN while implementing a U-Net generator with the traditional encoder/decoder depth of 3 [37]. Using a similar generator to that of Bass *et al.* [37], Ben-Cohen *et al.* were able to generate synthetic Positron Emission Tomography (PET) images from measured CT in a cGAN framework [38]. In the rest of this manuscript, we will use the model adopted by Ben-Cohen *et al.* as a benchmark for comparison and will refer to this model as U-cGAN. In our current application, the number of trainable parameters for U-cGAN is 4,868,614.

Our group introduced, for semantic segmentation, the sU-Net concept with an encoder/decoder depth of 2 [21]. Compared to the commonly used U-Net, sU-Net tends avoid unnecessary complexity by limiting the number of parameters to be optimized, avoiding the vanishing gradient effect, and requiring less training data. When supplemented by general anatomical topography knowledge, sU-Net performed accurate and rapid image segmentation for five structures on T2-weighted, TSE-SSH MR images of patients with cervical cancer [21]. Herein we propose the first use of the sU-Net model for image synthesis.

## III. THE PROPOSED sU-cGAN FRAMEWORK

**FIGURE 2.**
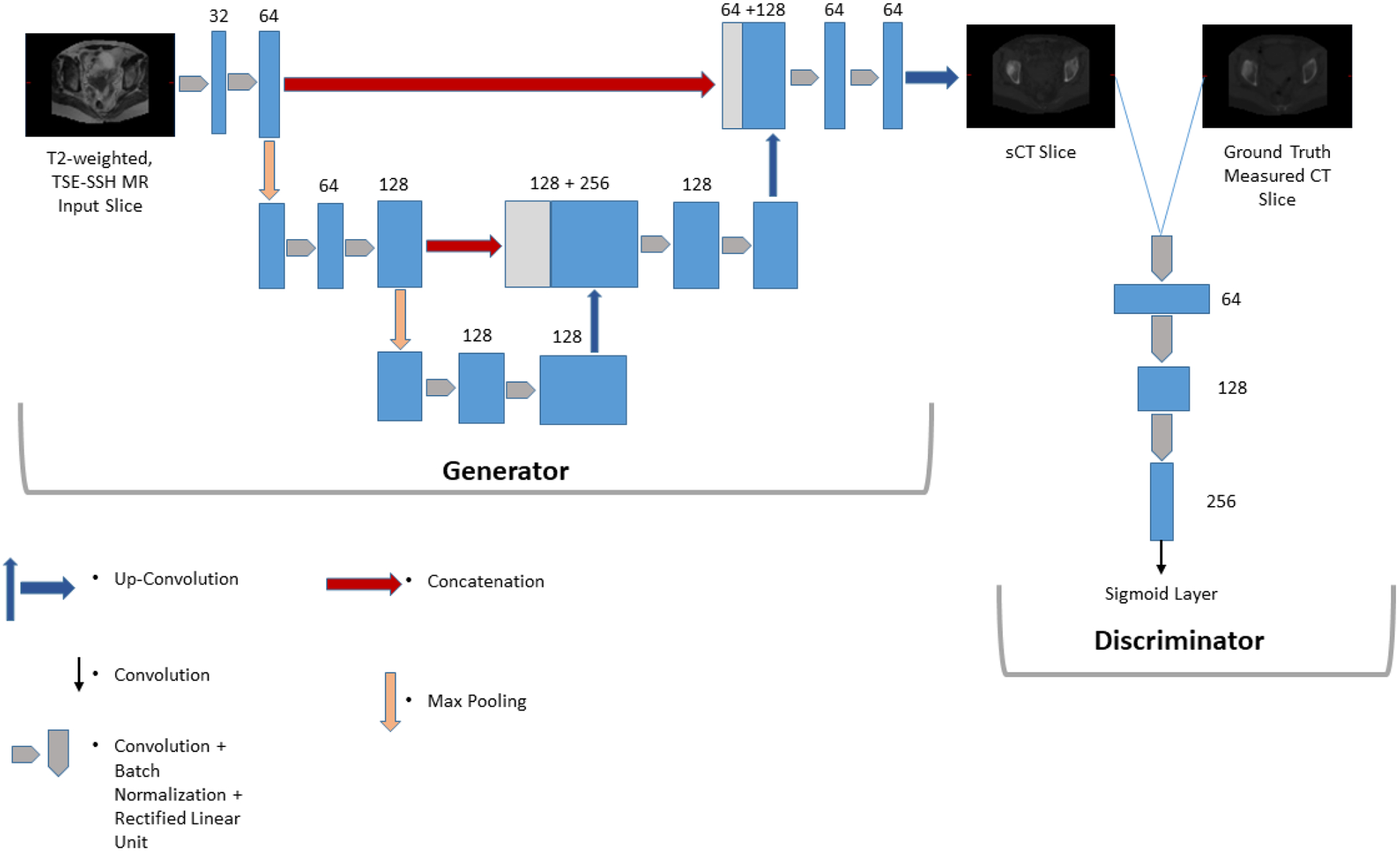
sU-cGAN structure. The number on the top/side of the boxes represent the number of features. The white boxes represent the concatenated feature maps.

The proposed sU-cGAN structure is illustrated in Fig. 2, and its number of trainable parameters is 3,163,142. The generator consists of an sU-Net that takes two-dimensional (2D) T2 weighted, TSE-SSH MR transverse image slices as a single channel input. The choice of this input derives from the FIGO recommendation explained in Section II.A, and would significantly and cost-effectively accelerate the workflow of the cervical cancer diagnosis, staging, and radiation therapy planning.

The sU-Net contracting and expanding pathways comprise the succession of convolutional [39], batch normalization [40], and rectified linear unit [41] layers. Convolutional layers filter their input by applying convolution kernels via the multiply-accumulate operation [42]. Batch normalization layers set the parameters mean and variance to 0 and 1, respectively, and thus enhancing convergence [43]. Rectified linear unit threshold values are at 0 by applying the activation function *f*_*ReLU*_ defined as:

(4) $$f_{\text{ReLU}}\left(x\right)=\text{max}\left(0,x\right)$$

Due to its non-saturating and linear form, the rectified linear unit accelerates further the gradient convergence and shortens the training time when compared to other activation functions such as hyperbolic tangent or sigmoid [44]. At each encoding stage in the descending branch, a 2 × 2 max pooling [45] layer scales down the size of the hidden layers while conserving an invariance to translations [46]. In contrast, an up-convolution layer is used at each decoding stage in the ascending branch, and thus projecting the feature maps into a higher dimensional space [47]. The ascending layers features are concatenated with the descending layers features, enabling sU-Net of improved pixel representation through the integration of high- and low-level features [48].

As for the discriminator, it consists of three blocks of the arrangement of convolutional [39], batch normalization [40], and rectified linear unit [41] layers, followed by a single convolution layer, and terminated by a sigmoid layer that scales the output to [0,1].

We incorporated in the sU-cGAN loss function the *L1* distance that is defined as follows:

(5) $$L\text{1}\left(G\right)\text{=}E_{x,\;y}\left[\left\|x-G\left(y\right)\right\|\right]$$

This approach is based on the previous experience with GAN frameworks where adding *L*1 yielded synthetic images close to the ground truth, with minor blurring at the edges ad negligible artifacts [31], [49]. Consequently, $L_{sU-\text{cGAN}}$ can be finalized as:

(6) $$L_{sU-\text{cGAN}}=L_{\text{cGAN}}\left(D,G\right)+\lambda L1\left(G\right)$$

where *λ* is a multiplicative factor that weights the contribution of *L1* into the sU-cGAN total loss.

## IV. EXPERIMENT

### A. DATA ACQUISITON

An IRB-approved study was conducted at University Hospitals Cleveland Medical center to retrospectively review the charts of adult female patients treated between June 2015 and June 2018 for a biopsy-proven cervical cancer. Among these patients, 11 had undergone a planning CT and PET/MR. PET/MR images were acquired using a Philips Ingenuity TF PET/MR system [50], [51], according to our institution protocol as follows: Field of view (FOV) of 300 mm, slice thickness of 4–5 mm, voxel size in the antero-posterior and left-right of 0.53–0.63 mm, TE of 80 milliseconds, TR of 1097 milliseconds, and a total scan duration of 60 seconds. T2-weighted, TSE-SSH MR Images were resampled to a pixel spacing of 3.2 mm × 3.2 mm × 5 mm and were manually contoured. The contouring process was described in detail elsewhere [21]. Five structures were identified: gross tumor volume (GTV); bilateral femurs; bladder; and anorectum. CT was acquired using a Philips Brilliance 16 multislice CT scanner (Cleveland, OH). Both MR and CT images were acquired using full bladder filling and three hours after fasting in order to decrease bowel peristalsis.

**FIGURE 3.**
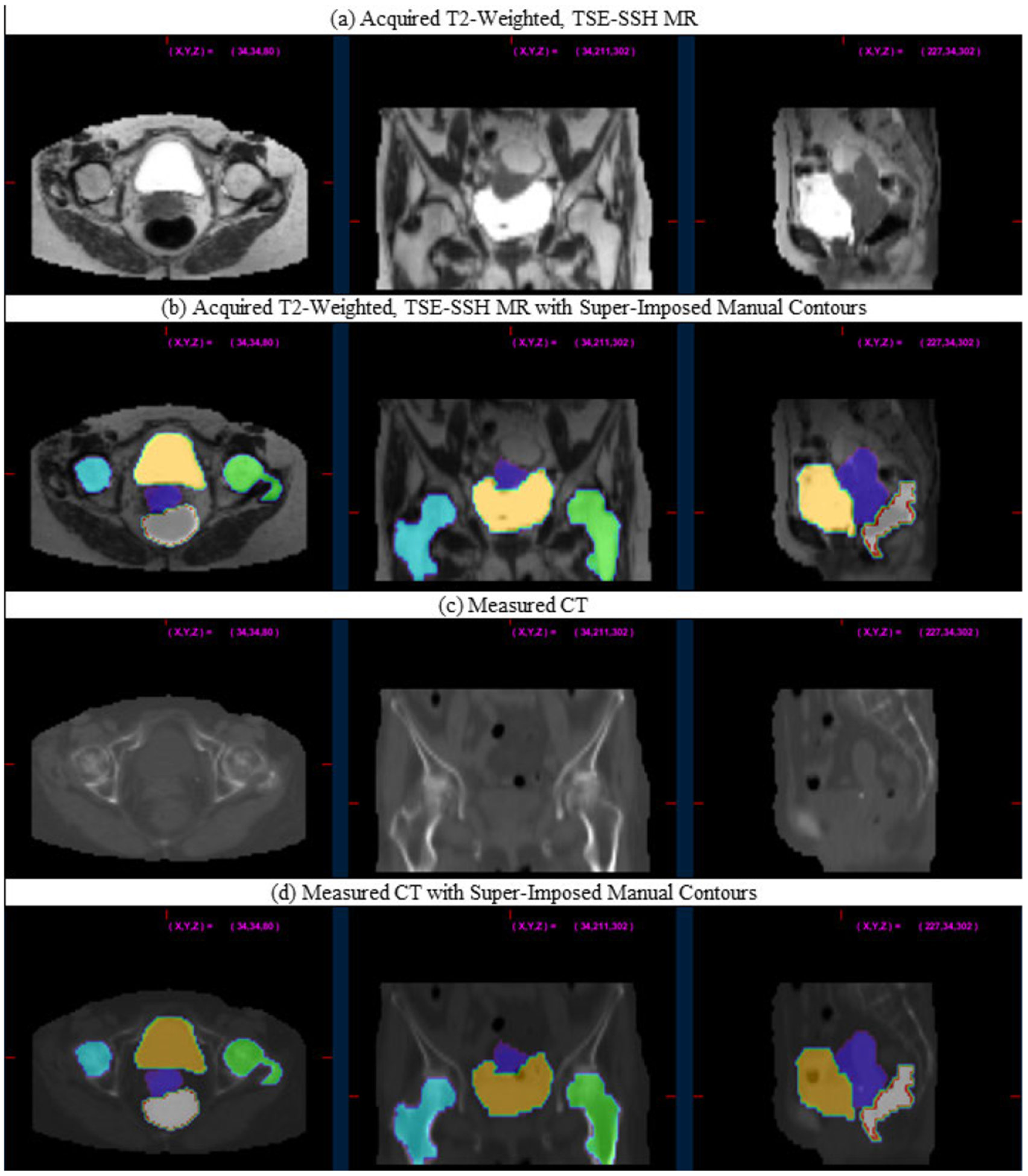
Axial, coronal, and sagittal (left to right) views of T2-weighted TSE-SSH MR (a), with the measured CT (b) after registration. Manual contours were initially drawn on the MR images (b), then projected to the CT images (d). GTV: Violet, Right Femur: blue, Left Femur: Green, Bladder: Orange, Anoerctum: Grey.

T2-weighted, TSE-SSH MR, and CT Images were de-identified and uploaded into MIM (MIM Software, Inc, Cleveland, OH). CT images were registered to the T2-weighted, TSE-SSH images using the MIM Reg Refine deformable image registration tool [52]–[55]. Images were then visually inspected and loaded into MATLAB 2020a (MathWorks, Inc.) using the COMKAT Image Tool [56], [57], and intensities were normalized to [0, 1] for training. Fig. 3 displays an example of CT, T2-weighted TSE-SSH MR images, and the manually delineated contours. Patient age, tumor histology, radiological FIGO stages, and final images size are summarized in Table 1.

### B. TRAINING AND PREDICTION

The weights in the convolutional and up-convolutional layers were initialized using He initializer [58] by sampling from a normal distribution where the mean is 0 and the variance is inversely proportional to the filter size and the channels number [58]. A leave-one-out training and validation method was adopted in our experimental design wherein 10 datasets are used for training, and the one remaining dataset is used for testing. Left/right flipping was used for data augmentation. Using MATLAB 2020a (MathWorks, Inc.), the experiment was performed on an Intel ®Xeon ®Silver 4116 CPU, 12 Cores, 24 Logical Processors, 2.10GHz, 128G RAM, NVIDIA TITAN XP, 24 GB GPU. The number of slices used for training and prediction at each experiment instance are listed in Table 2.

To highlight the performance of sU-cGAN, we also ran three separate experiences using U-cGAN [38], VGG16 [59] and ResNet [60]. The U-cGAN structure is described in III.C. It has the same loss function of sU-cGAN, but it differs from by its generator structure. VGG16 and ResNet are two commonly used networks in the field of image analysis and these have been detailed elsewhere [59], [60]. The minibtach size was 1, the maximum number of epochs was 200, and the Adam random gradient descent algorithm was used for loss function minimization [61]. $L_{sU-\text{cGAN}}$. was used as an objective function. As for the hyperparameter *λ*, an initial analysis of its effect had already been initiated by Isola *et al*. [31], and was subsequently adopted by different researchers in the medical imaging field [35], [37], [38]in which the choice of *λ* = 100 yielded the best results. In line with the previously published literature [31], [37], [38], we chose *λ* = *100* in the U-cGAN and sU-cGAN loss functions.

### C. PERFORMANCE EVALUATION

For each network, we recorded the training and prediction times. After visual inspection, we applied an air mask to the images in order to exclude air outside the body. We then reported for each sCT image volume the mean absolute prediction error (MAPE), the root mean square deviation and (RMSE) as defined in (7) and (8):

(7) $$\text{MAPE}=\frac{1}{N}\sum_{i=1}^{N}\left|HU_{CT}\left(i\right)-HU_{sCT}\left(i\right)\right|$$

(8) $$\text{RMSE}=\sqrt{\frac{\sum_{i=1}^{N}{\left(HU_{CT}\left(i\right)-HU_{sCT}\left(i\right)\right)}^{2}}{N}}$$

where *N* represents the total number of voxels in a given image volume and *HU*_*sCT*_ (*i*) and *HU*_*CT*_ (*i*) refer to the CT number, expressed in Hounsfield Units, of the voxel *i* in the sCT and measured CT, respectively. Furthermore, we also included in the evaluation metrics the peak-signal-to-noise ratio (PSNR) and the structural similarity index measure (SSIM). SSIM was originally introduced by Wang *et al.* [62] in 2004 as an objective metric that correlates with the perception of the human visual system. PSNR was originally introduced as a quality metric for video processing, then was adopted later for evaluation of medical images [63]. Mathematically, PSNR can be defined as follows:

(9) $$\text{PSNR}=10{\text{log}}_{10}\left(MAX^{2}/MSE\right)$$

where MAX represents the maximum intensity value, and MSE represents the mean square error between sCT and the measured CT. As for SSIM, it can be calculated as:

(10) $$\text{SSIM}\left(sCT,CT\right)=\frac{\left(2\mu_{sCT}\mu_{CT}+c_{1}\right)\left(2\delta_{sCT,CT}+c_{2}\right)}{\left(\mu_{sCT}^{2}+\mu_{CT}^{2}+c_{1}\right)\left(\delta_{sCT}^{2}+\delta_{CT}^{2}+c_{2}\right)}$$

where *μ*_CT_ and *μ*_sCT_ represent the CT and sCT mean HU respectively, *δ*_CT_ and *δ*_sCT_ correspond to the CT and sCT image HU variance respectively, and *δ*_sCT,CT_ corresponds to the HU covariance of the CT and sCT images. Finally, we plotted for each contour the CT HU histogram, and its overlay with the sCT histogram.

### D. RESULTS

**FIGURE 4.**
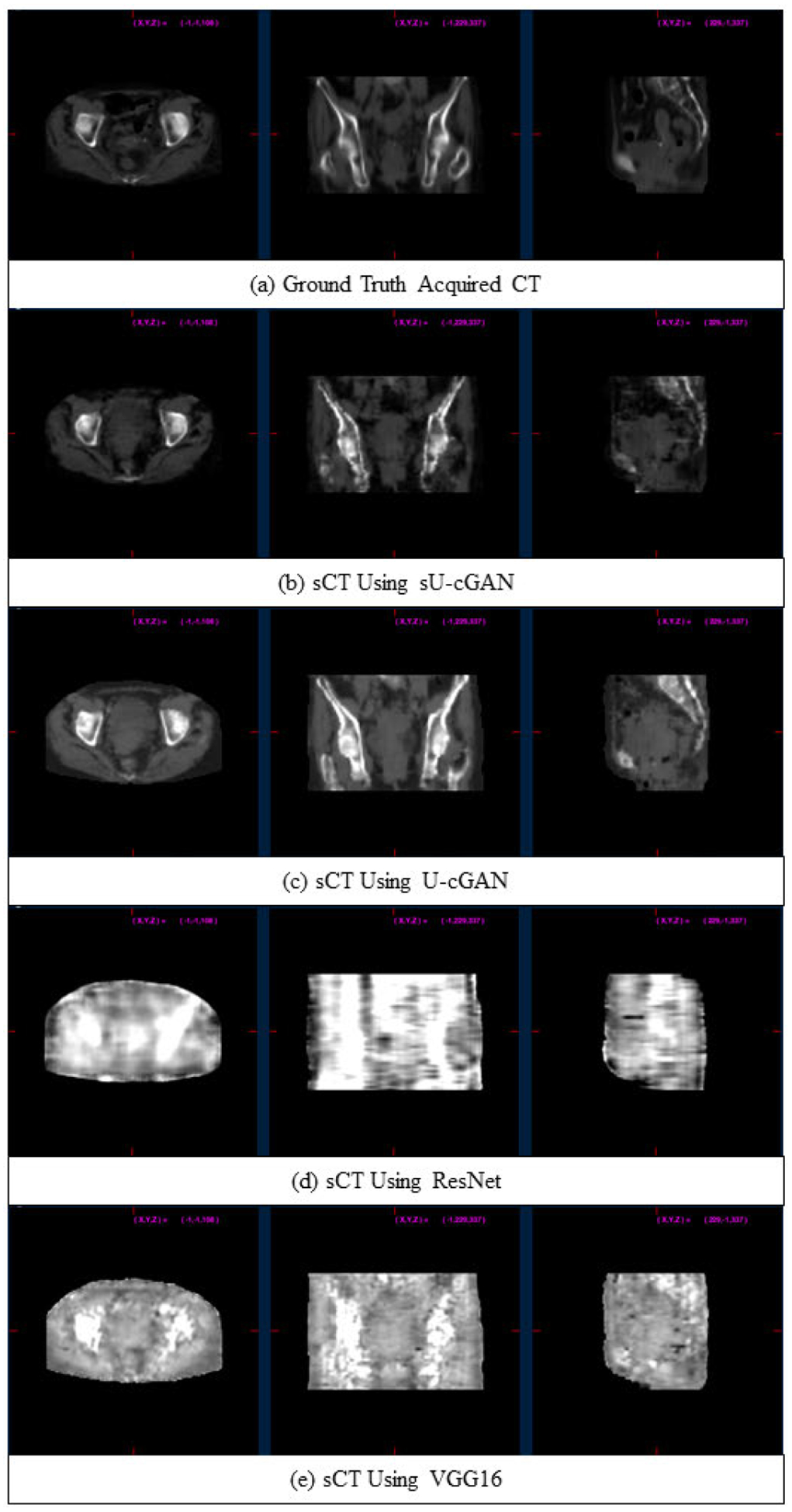
Axial, coronal, and sagittal (left to right) views of the measured CT (a), sCT using sU-cGAN (b), sCT using U-cGAN (c), sCT using ResNet (d), and sCT using VGG16 (e).

**FIGURE 5.**
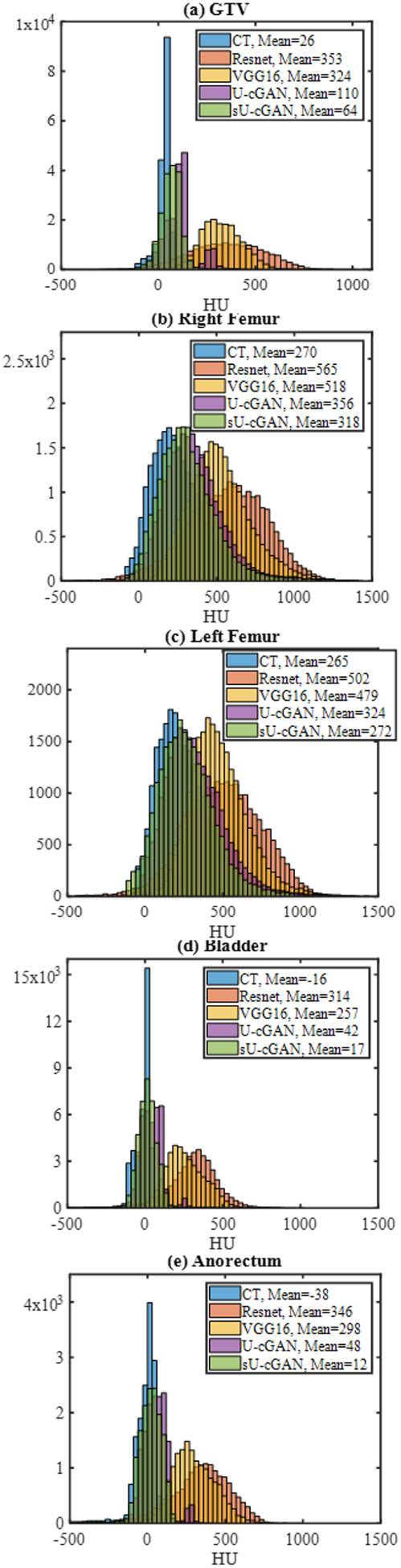
HU histograms of the GTV (a), right femur (b), left femur (c), bladder (d), and anorectum (e) for CT, sU-cGAN, U-cGAN, VGG16, and ResNet.

Example sCT, generated using sU-cGAN, U-cGAN, VGG16, and ResNet, are shown in Fig. 4. By visual inspection, the images obtained using VGG16 and ResNet are blurred and do not preserve any anatomical topography or internal organs’ gross structure. In contrast, images with sU-cGAN and U-cGAN maintain the overall anatomical topography as organs can be visually differentiated. The sU-cGAN appears more similar to the measured CT than U-cGAN, with bone being more intense on U-cGAN compared to the measured CT, and both sU-cGAN and U-cGAN showing some fault at the level of the bowels that should appear dark black. Table 3 displays the MAPE, RMSE, PSNR, SSIM, and training and prediction times with each of the four networks. In concordance with the visual findings, sU-cGAN and U-cGAN clearly outperformed VGG16 and ResNet in terms of MAPE, RMSE, PSNR, and SSIM. When comparing the cGAN frameworks head-to-head, sU-cGAN achieved the lowest mean MAPE (72.25 ± 25.42 with sU-cGAN vs. 99.64 ± 61.43 with U-cGAN) the lowest mean RMSE (115.74 ± 21.84 with sU-cGAN vs. 135.35 ± 53.95 with U-cGAN), the highest PSNR (63.41 ± 1.67 with sU-cGAN vs. 61.17 ± 2.20 with U-cGAN), and the highest SSIM (0.839 ± 0.044 with sU-cGAN vs. 0.823 ± 0.044 with U-cGAN). The overall superiority of the cGAN framework over VGG16 and ResNet, and of sU-cGAN over U-cGAN is also pronounced at the level of each contoured structure as displayed in Table 4, where sU-cGAN had the lowest MAPE and RMSE. Notably, sU-cGAN scored the lowest mean MAPE with the bladder (MAPE = 64.05 ± 22.55) and the lowest mean RMSE with the GTV (RMSE = 85.96 ± 42.17). In terms of tissue types, sU-cGAN seems to have better performance for soft tissue (GTV) and water-containing organs (bladder) than bone (right and left femur) and air-filled organs (anorectum). The sU-cGAN superior performance is further evident in the HU histograms findings displayed in Fig. 5, as the sU-cGAN HU distribution is the closest to the measured CT HU distribution. Interestingly, the sU-cGAN HU histograms of the left and right femurs (Fig. 5(b) and 5(c)), is more uniform than the GTV (Fig. 5(a)), bladder (Fig. 5(d)), and anorectum (Fig. 5(e)). By visual comparison, the HU histograms overlay is more similar to sU-cGAN than the three other networks. With respect to prediction time, the four networks achieved an extremely small prediction time ranging between 1.21 s (VGG16 for Subject 1) and 3.21 s (ResNet for subject 9). Numerically, VGG16 achieved the fastest training time and prediction time. The training times for ResNet and U-cGAN were in the range of 11 hours, while the training time for sU-cGAN was in the range of 7 to 8 hours.

## V. DISCUSSION

While conceptually based on an extensive use of diagnostic and therapeutic tools, precision medicine is nowadays challenged by the increased demand for cost-effective health care practice [64]. This challenge has been more pronounced lately with the current COVID-19 pandemic, in which extensive multi-modality imaging has become a luxury rather than a necessity. Under this perspective, in this study we introduced a novel workflow for sCT generation in patients with cervical cancer. The workflow complements our previously published research and enables - using a single, shortly timed MR sequence - the generation of sCT for PET/MR attenuation correction and radiation therapy planning by making use of automatic contouring and the generated sCT. Overall, the workflow integrates the current cost effective “reductionism” requirement, without jeopardizing the required precision for a patient-centered care. T2-weighted, TSE-SSH MR images can be easily and rapidly acquired across multiple platforms and thus do not usually present a source of discomfort for the patient. Therefore, the suggested approach addresses most of the inconveniences encountered in previous MR-based workflow. A particularly important outcome is that radiological diagnosis, PET/MR attenuation correction, automatic contouring, and radiation therapy planning can now be achieved based solely on T2-weighted TSE-SSH MR images. Not only is there no need for multiple MR acquisitions, we can now use the one MR acquisition that is routinely collected in the clinical workflow.

From a computational point of view, the vanishing gradient effect has been a drawback in deep learning since early 1990 [65]. While our workflow adopts the latest approaches in the field of computer vision by combining cGAN ftramework to U-Net structure, it also simplistically addresses the vanishing gradient impasse by using an sU-Net. Compared to the classic U-cGAN, sU-GAN decreases the number of trainable parameters by 1,705,472, or 35%. The approach of using a compact form of the commonly used U-Net thus far has been successful in image synthesis and semantic segmentation and should be further explored as it enabled an accurate (MAPE < 80 HU) and rapid (prediction time with sU-cGAN less than 3 s) sCT generation. While the training time reduction of approximately four hours (11h with U-cGAN vs. seven hours with sU-cGAN) might not be clinically relevant as the training is usually done offline, such difference further highlights the computational advantage of sU-Net.

From a general perspective, lower sCT error is expected to lead to higher accuracy in PET attenuation correction and radiation therapy dose planning. This being said, the relationship between the amplitude of the sCT error and the subsequent error inaccuracies in PET attenuation correction and radiation therapy dose planning is not predictable. This was previously studied by Fetty *et al.* who found no correlation between MAPE and the radiation therapy dosimetric plan evaluation [35]. Under this perspective, we focused the validation of our study on HU accuracy and the convenience of our method in the context of the usual diagnostic and therapeutic workflow in gynecological malignancy. Nonetheless, sCT quality is sensitive to the MR-CT registration accuracy of the training data. However, our manuscript is proposing the use of sCT for PET attenuation correction and radiation therapy planning and not for diagnostic purposes. As such, the dosimetric inaccuracies engendered by MR-CT registration defects would be of lower amplitude than those of the diagnostic inaccuracies. Nevertheless, the Reg Refine image registration tool has been validated previously for dosimetric applications in different clinical settings [52]–[55], including CT to T2-weighted MR images registration.

While the sCT error obtained with our proposed methodology (MAPE range of [49.18; 116.04], Median 65.53 HU) is comparable to the previously published articles, it is important to note that our method has the advantage of using as input the T2-weighted, TSE-SSH sequence used for diagnosis and staging. Not only is this highly significant as there is no extra acquisition time compared to methods that use specialized pulse sequences, there is also avoidance of the need for image registration between different MRI sequences. Such advantages are extremely useful for a method to be adopted into routine clinical practice in gynecological brachytherapy and adaptive radiotherapy in which time preservation is required and only a few MRI sequences can be acquired while the patient is on the treatment table. Furthermore, we must indicate that comparison of metrics among different studies is challenging, as comparisons are highly dependent on the number of the datasets, registration accuracy, and image voxel size and resolution. Given that many of the previously published articles have used U-cGAN-derived models in their studies, we would expect sU-cGAN to score better metrics when used on their datasets. Compared to T1-weighted and T2-weighted images, the use of T2-weighted, TSE-SSH images is expected to result in more accurate sCT generation, given the higher tissue geometric conformity with the TSE-SSH images than with T1-weighted or T2-weighted images.

The main limitations of the workflow are the number of datasets, the inclusion of the pelvic area only, and the absence of PET standardized uptake value validation. As such, future studies should be initiated at multiple levels. First, the generalizability to brain/head and neck, thorax, and abdomen. The success of sU-cGAN in the pelvic area that encompasses multiple soft-tissue structures belonging to the female reproductive system and in close proximity to the lower digestive system, predicts a similar success in sCT generation for other anatomical areas. Moreover, the choice of 2D architecture in this study proceeded mainly from the limited data availability. Additionally, the results can be greatly improved if more data is acquired, as our system can be upgraded to consider spatial relationships in three dimensions using 3D sU-Net. Finally, this work can be directly applied in adaptive radiotherapy in which a planning MR is acquired immediately before each radiation therapy plan. By acquiring T2-weighted TSE-SSH images the acquisition time is reduced, and by applying our contouring algorithm, planning is automated and physician time expenses are reduced.

## VI. CONCLUSION

A convenient method for sCT generation was presented in this article, using a single MR sequence and an sU-Net as the generator of a cGAN. Despite the simplified generator architecture, the anatomically complex female pelvic structure, and the limited available data, sU-cGAN was able to generate, in less than 3 s, an accurate sCT volume with MAPE < 80 HU. The results are comparable to those obtained by Maspero *et al.* [33], with the exception that Maspero *et al.* used 32 sets of MR-CT pairs for training and the used input consisted of Dixon sequences [33].

Given the TSE-SSH advantages, this method can generally be used for PET/MR attenuation correction and radiation therapy planning as it dramatically facilitates the automation of the PET/MR and MR-only based cervical cancer treatment conventional and adaptive workflows. Being routinely collected for cervical cancer staging, the use of TSE-SSH as input for sCT generation requires no additional cost or time for data collection. Therefore, results can be further optimized by acquiring more data, in both retrospective and prospective studies. As for the sU-Net structure, our work for semantic segmentation and image synthesis underscores the potential of a simplified network structure to perform difficult computer vision tasks when manipulated appropriately by the human user. The results demand attention to reconsider an essential paradigm in deep learning: “How deep is too deep?” [66].

## Supplementary Material

supp1-3049781

## Figures and Tables

**TABLE 1.** Patients’ age, tumor’s histology, radiological FIGO stages, and final images size.

Subject Number	Age at Diagnosis (Y)	Histology	FIGO Radiological Staging	MR and CT Size after Registration (voxels)
1	79	SCC	IIIB	144×144×50
2	81	SCC	IIB	144×144×50
3	70	SCC	IVB	144×144×50
4	79	Adeno	IIB	144×144×44
5	57	SCC	IIIB	144×144×50
6	82	SCC	IIB	144×144×44
7	62	SCC	IIB	144×144×44
8	49	SCC	IIB	144×144×44
9	52	Mixed	IIA	144×144×44
10	45	SCC	IIB	144×144×44
11	83	Adeno	IB	144×144×44

SCC = Squamous Cell Carcinoma, Adeno = Adenocarcinoma, Mixed = Mixed SCC + Adeno

**TABLE 2.** Training and prediction sample size at each experiment instance.

Experiment Instance	Number of 2D Slices Used for Traning	Number of 2D Slices Used for Prediction
1	458	50
*2*	458	50
*3*	458	50
*4*	464	44
*5*	458	50
6	464	44
7	464	44
*8*	464	44
*9*	464	44
10	464	44
11	464	44

**TABLE 3.** Performance Metrics for each of the trained networks.

Subject	Network	1	2	3	4	5	6	7	8	9	10	11	Mean±SD
MAPE	sU-cGAN	**52.55**	**78.88**	65.53	**65.35**	106.03	**43.78**	**104.91**	49.18	50.89	**67.97**	116.04	**72.83±25.42**
	U-cGAN	67.56	111.87	**58.95**	88.32	**100.63**	99.12	266.88	**41.19**	**43.45**	103.88	**114.16**	99.64±61.43
	Vgg16	255.76	249.8	479.26	270.7	408.97	351.76	409.2	296.57	220.53	181.42	330.51	314±91.16
	ResNet	397.13	463.49	346.06	448.01	333.72	415.53	310.80	402.72	298.20	405.29	387.39	382.58±53.9
RMSE	sU-cGAN	107.41	**112.29**	121.15	**110.53**	146.92	**89.1**	**152.20**	84.94	107.04	**104.27**	137.29	**115.74±21.84**
	U-cGAN	**106.22**	138.76	**117.10**	122.44	**139.8**	133.01	289.64	**84.64**	**100.41**	124.9	**131.95**	135.35±53.95
	Vgg16	276.48	273.13	497.28	296.48	434.67	372.05	438.50	315.96	255.21	210.99	350.14	338.26±89.05
	ResNet	441.08	503.26	398.36	491.7	402.30	452.86	385.04	449.18	348.90	440.0	432.0	431.33±45.43
PSNR	sU-cGAN	61.03	**62.78**	61.31	**63.37**	61.79	**66.32**	**64.39**	**64.52**	65.93	**63.50**	62.58	**63.41±1,67**
	U-cGAN	**61.82**	61.03	**61.52**	62.63	**62.17**	61.92	56.87	64.11	**66.47**	62.35	**63.00**	62.17±2.20
	Vgg16	53.61	55.04	47.82	54.00	52.25	52.06	52.80	52.97	56.23	57.48	53.78	53.46±2.39
	ResNet	49.34	49.48	50.59	49.41	54.84	50.43	55.99	49.71	53.58	50.62	52.21	51.47±2.24
SSIM	sU-cGAN	**0.780**	**0.808**	0.736	**0.852**	0.878	**0.870**	**0.878**	0.831	0.869	**0.869**	0.855	**0.839±0.044**
	U-cGAN	0.747	0.779	**0.747**	0.819	**0.881**	0.794	0.848	**0.854**	**0.892**	0.830	**0.857**	0.823±0.048
	Vgg16	0.557	0.685	0.528	0.678	0.771	0.682	0.784	0.620	0.692	0.723	0.764	0.680±0.080
	ResNet	0.514	0.621	0.513	0.638	0.743	0.660	0.754	0.601	0.667	0.675	0.725	0.646±0.078
Training Time	sU-cGAN	7 h 47.99 min	7 h 36.92 min	7 h 43.75 min	7 h 58.05 min	7 h 37.88 min	7 h 4.15 min	7 h 54.25 min	7 h 26.38 min	7 h 32.27 min	8 h 3.22 min	7 h 50.15 min	7 h 44.91 min± 11.19 min
	U-cGAN	11 h 1.69 min	10 h 53.43 min	10 h 59.47 min	10 h 41.53 min	10 h 59.77 min	11 h 1.118 min	10 h 43.53 min	10 h 50.31 min	11 h 59.34 min	11 h 51.81 min	11 h 19.20 min	11 h 7.39 min ± 25.94 min
	Vggl6	**2 h 43.18 min**	**2 h 58.79 min**	**2 h 45.5 min**	**2 h 46.32 min**	**2 h 52.2 min**	**2 h 51.5 min**	**2 h 52.37 min**	**2 h 48.4 min**	**2 h 41.23 min**	**2 h 47.34 min**	**2 h 50.1 min**	**2 h 48.82 min ± 4.81 min**
	ResNet	11 h 33.48 min	11 h 44.21 min	11 h 30.1 min	11 h 26.51 min	11 h 25.02 min	11 h 21.14 min	11 h 5.4 min	11 h 10.67 min	11 h 41.93 min	11 h 48.97 min	11 h 41.54 min	11 h 29.9 min ± 13.94 min
Prediction Time (s)	sU-cGAN	2.07	1.97	1.98	1.78	1.98	1.96	1.83	1.69	1.76	1.88	1.86	1.89 ± 0.11
	U-cGAN	2.94	2.78	2.84	2.33	2.97	2.70	2.5	2.34	2.99	2.73	2.44	2.69 ± 0.25
	Vgg16	**1.21**	**1.32**	**1.32**	**1.26**	**1.40**	**1.33**	**1.27**	**1.32**	**1.24**	**1.35**	**1.25**	**1.3 ± 0.06**
	ResNet	2.26	2.27	2.31	2.37	2.99	2.2	2.41	2.27	3.21	2.29	3.54	2.55±0.46

The best value achieved for each of the performance metrics (lowest for MAPE, RMSE, Training, and Prediction times, and highest for PSNR and SSIM) are highlighted in bold.

**TABLE 4.** Performance Metrics for each contour.

Metric	Network	GTV	Right Femur	Left Femur	Bladder	Anorectum
MAPE	sU-cGAN	**62.15 ± 24.59**	**146.1 ± 36.1**	**139.43 ± 36**	**64.05 ± 22.55**	**108.9 ± 51.29**
	U-cGAN	91.12±62.27	167.09 ± 68.60	151.65 ± 56.73	85.93 ± 5827	131.45 ± 76.07
	Vggl6	293.41 ± 89.04	303.62 ± 61.29	276 ± 53.65	283.28 ± 110.04	348.95 ± 110.42
	ResNet	42.69 ± 12.89	273.8 ± 72.18	296.28 ± 62.33	40.38 ± 21.34	69.97 ± 40.07
RMSE	sU-cGAN	**85.96 ± 42.17**	**194.47 ± 50.39**	**191.83 ± 54**	**88.17 ± 38.94**	**181. 9 ± 94.93**
	U-cGAN	109.73 ± 66.52	210.23 ± 75.05	197.12 ± 67.74	108.04 ± 61.7	191.19 ± 98.1
	Vgg16	307. 31 ± 85	359.1 ± 58.92	326.97 ± 53.71	298.56 ± 108.84	384.18 ± 119.28
	ResNet	62.98 ± 45.09	334.84 ± 71.14	329.68 ± 62.5	71.26 ± 44.48	130.08 ± 93.27
SSIM	sU-cGAN	0.07±0.06	**0.51±0.16**	0.48±0.21	**0.12±0.10**	**0.06±0.08**
	U-cGAN	**0.07±0.07**	0.50±0.17	**0.52±0.18**	0.12±0.11	0.01±0.08
	Vgg16	0.01±0.01	0.08±0.05	0.11±0.06	0.02±0.03	−0.02±0.02
	ResNet	0.00±0.01	0.03±0.03	0.06±0.05	0.01±0.01	0.00±0.01
PSNR	sU-cGAN	**14.13±4.37**	**29.30±1.48**	**28.12±1.77**	**18.71±3.59**	12.57±4.54
	U-cGAN	10.89±3.35	27.93±1.98	27.09±2.79	17.94±5.48	10.51±3.55
	Vgg16	12.58±2.64	18.68±2.58	18.58±2.19	12.89±1.68	**12.97±1.48**
	ResNet	12.52±1.45	15.22±1.25	15.44±1.95	12.22±1.72	11.85±1.28

The best value achieved for each of the performance metrics (lowest for MAPE, RMSE, Training, and Prediction times, and highest for PSNR and SSIM) are highlighted in bold.
