# Supplementary figures and images for: Synthetic CT Generation of the Pelvis in Patients With Cervical Cancer: A Single Input Approach Using Generative Adversarial Network
Source: IEEE Access. Author manuscript; Available in PMC 2021 Mar 19. (PMC7978399; doi:10.1109/access.2021.3049781)

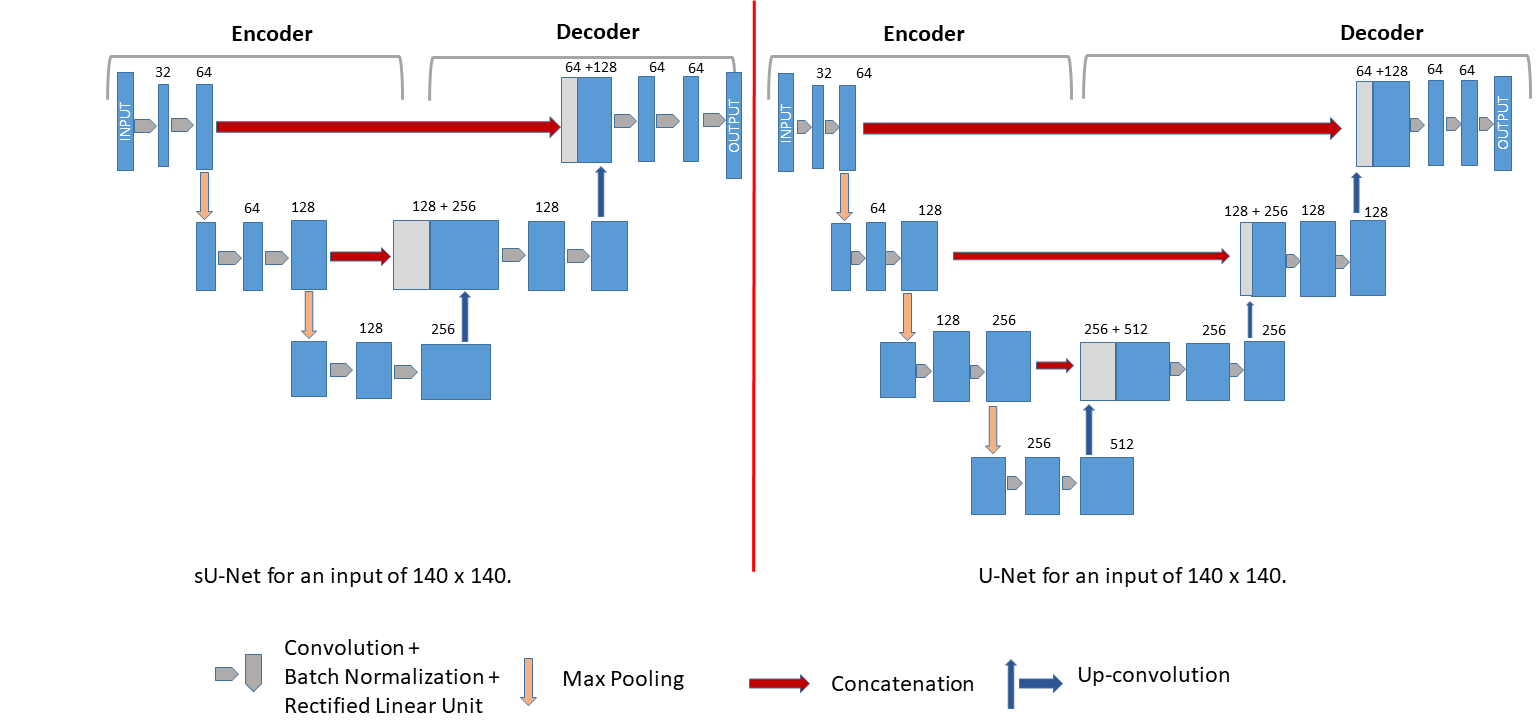

Supplement: supp1-3049781 [file NIHMS1668281-supplement-supp1-3049781.tif]
